# Surgical repair for aortic dissection accompanying a right-sided aortic arch

**DOI:** 10.1186/1749-8090-5-35

**Published:** 2010-05-10

**Authors:** Yukio Obitsu, Nobusato Koizumi, Toru Iwahashi, Naozumi Saiki, Hiroshi Shigematsu

**Affiliations:** 1 Department of Vascular Surgery, Tokyo Medical University 6-7-1 Nishishinjuku, Shinjuku-ku, Tokyo, Japan 160-0023

## Abstract

Aortic anomaly in which a right-sided aortic arch associated with Kommerell's diverticulum and aberrant left subclavian artery is rare. The present report describes a patient with type-B aortic dissection accompanying aortic anomalies consisting of right-sided aortic arch and the left common carotid and left subclavian artery arising from Kommerell's diverticulum. As dissecting aortic aneurysm diameter increased rapidly, Single-stage surgical repair of extensive thoracic aorta was performed through median sternotomy and right posterolateral fifth intercostal thoracotomy, yielding favorable results. Our surgical procedures are discussed.

## Background

Aortic anomalies in which a right-sided aortic arch is complicated by an aberrant left subclavian artery are rare, with a reported incidence of 0.05% of the population [1]. While many patients are asymptomatic, surgery is indicated when accompanied by Kommerell's diverticulum aneurysm or vascular ring compression. We performed single-stage surgical repair of extensive thoracic aorta for a patient with aortic anomalies consisting of right-sided aortic arch, the left common carotid and left subclavian artery arising from Kommerell's diverticulum and type B aortic dissection, obtaining favorable results. Our surgical procedures are reported herein.

## Case presentation

**Figure 1:**
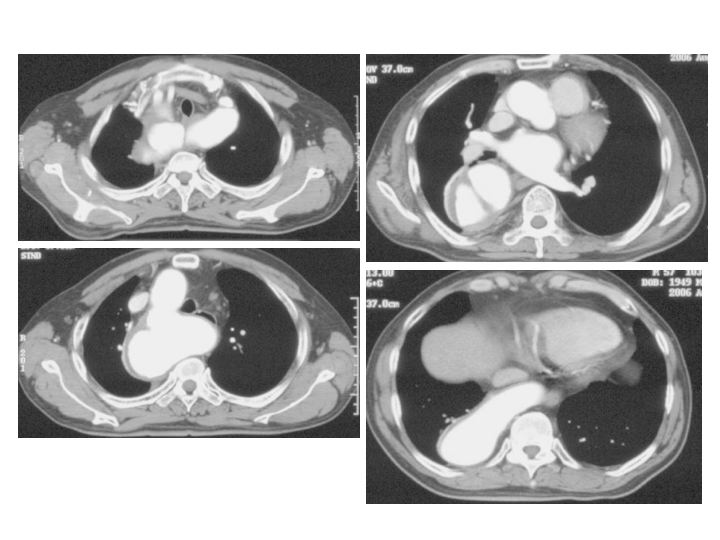
Contrast-enhanced CT scan demonstrating the dissecting aneurysm associated with the right aortic arch and left subclavian, common carotid artery from Kommerell's diverticulum.

**Figure 2:**
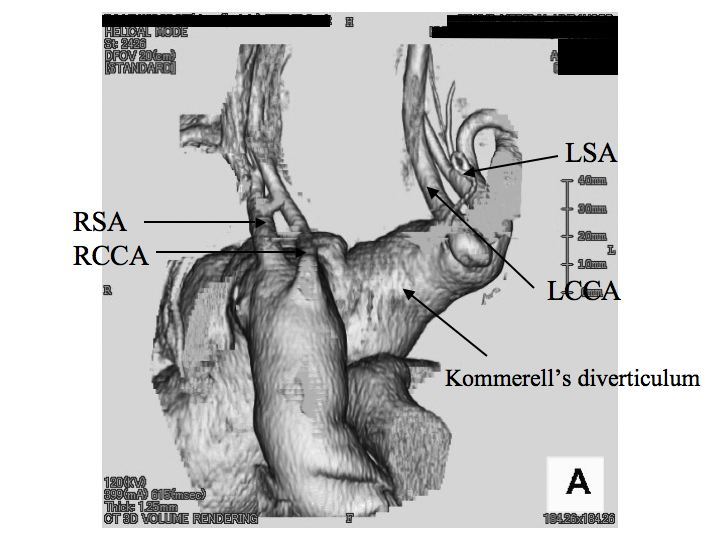
3D CT scan showing the Kommerell's diveticulum and the anomalous pattern of the arch branches.

The patient was a 57-year-old man who had been diagnosed with right-sided aortic arch and Kommerell's diverticulum. Since he was asymptomatic at diagnosis, he was being monitored. The patient developed type B acute aortic dissection (DeBakey IIIa), and the dissecting aortic aneurysm diameter rapidly increased. He was referred to our hospital 4 months after onset of type B acute aortic dissection. Multidetector-row computed tomography (CT) showed right-sided aortic arch, an anomaly in which the left common carotid and left subclavian artery originated from Kommerell's diverticulum, and dissecting aortic aneurysm with a maximum diameter of 60 mm. Surgery was indicated (Figs. 1, 2). Although thoracic endovascular aneurysm repair was considered, single-stage surgical repair of extensive thoracic aorta was selected because the proximal landing zone was short and aortic curvature was severe.

**Figure 3:**
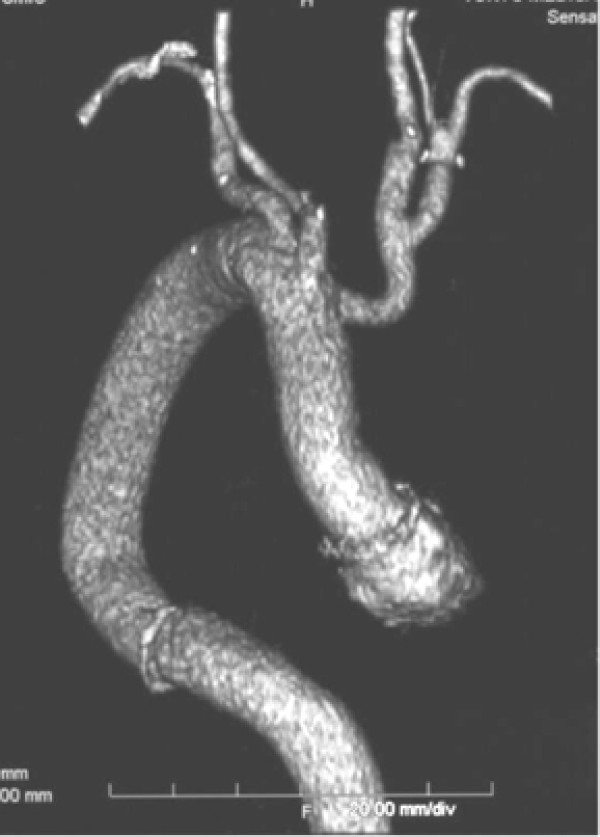
Postoperative 3D CT scan indicating successful repair of the total thoracic aorta

In surgery, the aneurysm was approached through median sternotomy and right posterolateral fifth intercostal thoracotomy, and extracorporeal circulation was established by infusing blood through the ascending aorta and right femoral artery and draining blood from the right atrium. Kommerell's diverticulum was on the posterior side of the esophagus, with the left common carotid and subclavian artery branching out on the left side of the trachea. Circulation was arrested at a core temperature of 26°C, and the aorta was dissected. The brain was protected by selective cerebral perfusion where a blood delivery cannula was inserted into the right common carotid artery, right subclavian artery, left common carotid artery and left subclavian artery. The aorta was clamped at the periphery of the right subclavian artery, and systemic circulation was resumed by infusing through the femoral artery. The proximal side was anastomosed using a 24-mm Intergard with four branches (St. Jude Medical, St. Paul, MN). The branches for reconstructing the left common carotid and subclavian artery were prepared using the lateral branch, and the left common carotid artery, left subclavian artery, right common carotid artery and right subclavian artery were reconstructed in that order. At this stage, blood was perfused from the first branch to maintain coronary and cerebral circulation. The aorta was clamped immediately above the diaphragm, a distal anastomosis was performed, and the Kommerell's diverticulum inlet was closed from inside the aorta. The duration of myocardial ischemia and extracorporeal circulation was 9 min and 152 min, respectively (Fig. 3). The postoperative course was favorable, and the patient was discharged without any complications. He is doing fine as of 4 years after surgery.

Surgery is not necessarily indicated for Kommerell's diverticulum accompanying aortic anomaly. However, if clinical symptoms are present related to vascular ring, such as respiratory and swallowing impairments, surgery is indicated for aneurysm formation because of the risk of peripheral embolism, aortic dissection and rupture associated with Kommerell's diverticulum [1,2]. Although the patient was asymptomatic, surgery was indicated because of Kommerell's diverticulum, aortic dissection and aneurysm diameter enlargement. Surgery for descending aortic aneurysm accompanying right-sided aortic arch has only been described sporadically [3,4]. Hybrid procedures combining with thoracic endovascular aneurysm repair and bypass has been reported in recent years [5,6]. Although we considered hybrid procedure, we decided on single-stage surgical repair of extensive thoracic aorta because cervical branch bypass was required, the proximal landing zone peripheral to the right subclavian artery was short, and aortic curvature was severe.

## Conclusion

With regard to surgery, unnecessary procedures could be omitted by improving branch anastomosis of the 4-branch artificial vessel, shortening the duration of myocardial ischemia and brain perfusion. In cases with aortic anomalies like the present patient, careful examination of surgical procedures on an individual basis is important.

## Consent

Written informed consent was obtained from the patient for publication of this case report and accompanying images. A copy of the written consent is available for review by the Editor-in-Chief of this journal.

## Competing interests

The authors declare that they have no competing interests.

## Authors' contributions

YO carried out the study design, data analysis and writing, NK, TI, NS and HS performed data collection. All authors read and approved the final manuscript.
